# Vaginal delivery: how does early hospital discharge affect mother and child outcomes? A systematic literature review

**DOI:** 10.1186/s12884-017-1465-7

**Published:** 2017-09-06

**Authors:** Nadia Benahmed, Lorena San Miguel, Carl Devos, Nicolas Fairon, Wendy Christiaens

**Affiliations:** KCE Belgian Health Care Knowledge Centre, Boulevard du Jardin Botanique 55, 1000 Bruxelles, Belgium

**Keywords:** Postpartum, Early discharge, Delivery, Mother, Children

## Abstract

**Background:**

There is an international trend to shorten the postpartum length of stay in hospitals, driven by cost containment, hospital bed availability and a movement toward the ‘demedicalization’ of birth. The aim of this systematic literature review is to determine how early postnatal discharge policies from hospitals could affect health outcomes after vaginal delivery for healthy mothers and term newborns.

**Methods:**

A search for systematic reviews, meta-analyses, and primary studies was carried out in OVID MEDLINE, Embase, CINAHL, Econlit and the Cochrane Library (Cochrane Database of Systematic Reviews, DARE and HTA databases). The AMSTAR checklist was used for the quality appraisal of systematic reviews. The quality of the retrieved studies was assessed by the Cochrane Collaboration’s tools. The level of evidence was appraised using the GRADE system.

**Results:**

Seven RCTs and two additional observational studies were found but no comprehensive economic evaluation. Despite variation in the definition of early discharge, the authors of the included studies, concerning early discharge and conventional length of stay, reported no statistical difference in maternal and neonatal morbidity, maternal and neonatal readmission rates, infant mortality, newborn weight gain, neonatal hyperbilirubinemia, or breastfeeding rates. The authors reported conflicting results regarding postpartum depression and competence of mothering, ranging from no difference according to length of stay to better results for early discharge. The level of evidence of the vast majority of outcomes was rated as low to very low.

**Conclusions:**

Because of the lack of robust clinical evidence and full economic evaluations, the current data neither support nor discourage the widespread use of early postpartum discharge. Before implementing an early discharge policy, Western countries with longer length of hospital stay may benefit from testing shorter length of stay in studies with an appropriate design. The issue of cost containment in implementing early discharge and the potential impact on the current and future health of the population exemplifies the need for publicly funded clinical trials in such public health area. Finally, trials testing the range of out-patient interventions supporting early discharge are needed in Western countries which implemented early discharge policies in the past.

## Background

In Western countries, there is a trend to shorten the postpartum length of stay in hospital driven by cost containment, hospital bed availability and a movement toward ‘demedicalisation’ of childbirth [1]. Although this trend was initiated as early as the 1940s in the United States [2] other countries followed later. Thus, while the average postpartum length of stay in Swedish hospitals was 6 days in the 1970s [3] it declined to 2.3 days in 2010 [4], reflecting a similar duration to that observed, the same year, in the Netherlands (2.1 days), New-Zealand, Ireland and the United States (2.0 days) [4]. In addition to shorter hospital stays, early postpartum discharge for healthy mothers and newborns was introduced to promote a more family-centred approach to birth allowing greater involvement of fathers, less sibling rivalry, improved rest and sleep for the mother, less exposure of the dyad mother-newborn to nosocomial infections, enhancement of maternal confidence in caring for the baby, and finally, less conflicting advice on breastfeeding [5]. However, concerns about early discharge arose regarding potential adverse outcomes such as delays in detecting and preventing maternal morbidities and neonatal pathologies [6], earlier weaning, lack of professional support, higher prevalence of postpartum depression and increased hospital readmissions for both mothers and infants [5]. Societal concerns about maternal and newborn welfare in the context of early discharge led the United States to vote the Newborns’ and Mothers’ Health Protection Act in 1996 making it mandatory for health insurers to cover postpartum hospitalisation for a minimum of 48 h after vaginal delivery and 96 h after a caesarean section [2]. Therefore, early discharge after vaginal birth is considered as less than 48 h in the United States. However, to date there is no standard definition of early postpartum discharge because of large variations in the usual average length of stay for vaginal delivery between countries (i.e. in 2011, the mean length of stay for single spontaneous delivery was 5.2 days in Hungary, 4.2 in France, 4.0 in Belgium, 2.8 in Australia, 1.7 in Canada and 1.5 in the United Kingdom) [4]. Consequently, early postpartum discharge varies from 12 to 72 h depending on the country [7].

As in some other Western countries, Belgium experiences an average hospital stay for vaginal deliveries of 4 days. Decision makers willing to introduce earlier hospital discharge need evidence based information about the effect of such policies on neonatal and maternal health outcomes. Therefore, this literature systematic review aims to determine how, early discharge policies with or without home care (support) affect health outcomes after a vaginal birth for healthy mothers and term newborns (≥ 37 weeks) in Western countries.

## Method

### Eligibility and search strategy

Studies included in this systematic review compare ‘early discharge of healthy mothers and newborns after a vaginal birth with or without home care’ with standard length of hospital stay as defined in the time and place where the studies were conducted.

Caesarean sections necessitate longer hospital stays and more complicated postnatal care. Therefore, we selected all studies that included vaginal birth of live singleton term infants with or without instrumental assistance. All studies must include hospital deliveries overseen by a health care professional in Western countries. Studies describing deliveries by caesarean sections, the presence of postpartum haemorrhage, substance abuse during the pregnancy, disability, domestic violence, low birth weight, preterm baby, or home birth were excluded.

Women’s views about length of stay or satisfaction with care are not included in this review because these outcomes are highly context-specific. As international comparisons are influenced by a wide range of confounders such as time, subjective wellbeing, type of care organisation… [8] and because of the lack of information regarding these confounders in the publications, the adjustment is challenging and will not be reviewed here.

This review was conducted according to the PRISMA statement [9]. The search for systematic reviews, meta-analyses, and primary studies was carried out by an information specialist (NF) and validated by other co-authors in OVID MEDLINE, Embase, CINAHL, Econlit and the Cochrane Library (Cochrane Database of Systematic Reviews, DARE and HTA database). MESH terms and related keywords were used in an extensively searched (detailed search strategies by databases are available in Additional file 1). Two independent researchers (NB, LS) performed the selection, the quality appraisal, and the data extraction of the studies. During the selection process, a Cochrane review of early postnatal discharge from hospital for healthy mothers and term infants was found [5]. This high quality review (see AMSTAR evaluation in Additional file 1) included only randomised controlled trials (RCTs) in a broader population than our population of interest. Therefore, relevant evidence was extracted from this review [5] to focus on our specific research question. In addition, the update presented here considered all comparative studies whatever the design, published until August 2015. Non-randomised studies were included only if they described an outcome that was not previously described in a RCT. Because health care organisations are perpetually evolving, we limited our search to new outcomes supported by non-randomised comparative studies during the update period.

The health outcomes under consideration were mortality, morbidity, hospital readmission rates for both mother and child. Additional neonatal outcomes (jaundice and weight gain) and maternal outcomes (counselling and psychological function). Breastfeeding rates were also studied.

### Assessment of methodological quality of evidence

The AMSTAR checklist was used for the quality appraisal of the systematic reviews [10]. The quality appraisal of RCTs and non-randomised studies was performed using the "Cochrane Collaboration’s tool for assessing risk of bias" [11].

### Statistical analyses

Categorical data were presented using risk ratios (RR) with 95% confidence intervals (95% CI). The mean difference with 95% CI was used to report continuous data. Statistical analyses for pooling intervention effects were performed using Review Manager 5.3®.

### Level of evidence

Level of evidence was categorized in 4 levels (high, moderate, low and very low) according to the GRADE system [12].

## Results

### Results of the search

From the Cochrane review, ten RCTs were extracted for full text examination. From those, three trials were excluded because deliveries by caesarean section were included and results were not reported separately for vaginal deliveries [13–15].

During the update process, no systematic reviews, meta-analyses, or RCTs were found. After a first sifting based on title and abstract, 16 non-randomised studies were retrieved for further analysis on full text [1, 2, 6, 16–28]. Finally, 2 non-randomised studies were included in our review [16, 27].

**Fig. 1:**
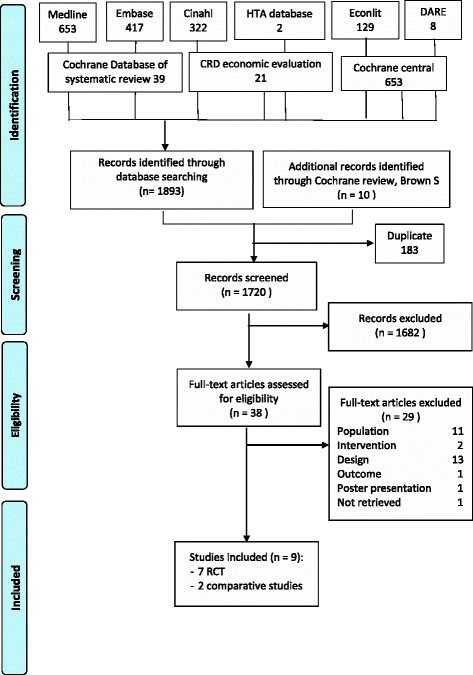
PRISMA flow diagram

The present systematic review includes 5 RCTs and two non-randomised studies (see Fig. 1 – Flow chart). Characteristics of the included studies are reported in Table 1. A list of excluded studies is provided in Additional file 1.

**Table 1:** Characteristics of included studies

Study	Design	Inclusion criteria	Intervention (n)	Control (n)	Age	Findings
Carty 1990 [29], Canada	RCT	Vaginal birth, prenatal classes attendance, stable relationship with partners	Group 1: discharge from 12 h to 24 h + nurse home visit on days 1, 2, 3, 5 and 10 (44) Group 2: discharge from 25 h to 48 h + nurse home visit on days 3, 5 and 10 (49)	Group 3: discharge at 4 days + nurse home visit on 10 (38)	Mean (SD) 30.24 (3.80)	Maternal outcomes ▪ Morbidity Problems requiring physician referral in the first 10 days postpartum *Intervention*: 5/93 (urinary tract infection, episiotomy infection, mastitis, subinvolution) *Control*: 3/38 (endometritis, episiotomy, subinvolution) ▪ Hospitalization within the first 1 month *Intervention*: 1/93 (urinary tract infection) *Control*: 1/38 (endometritis) ▪ Psychological functioning *Depressive affect at 1 month (Beck Depression Index)* Significant difference between group 2 and group 3 in favour of group 2 (*p* < 0.05) *Confidence (subscale of a questionnaire for women during their childbearing years)* At 1 week: score in favour of group 1 in comparison with group 3 (*p* < 0.03), no significant difference between group 1 and 2 At 1 month: no difference between groups Neonatal outcomes ▪ Morbidity Problems requiring physician referral in the first 10 days postpartum *Intervention*: 4.3% (hyperbilirubinemia and cord infection,) *Control*: 2.6% (ABO incompatibility and diaper rash) Breastfeeding duration *Intervention*: 87% *Control*: 79% Length of stay: mean in days (SD) Group 1: 1.12 (0.40) Group 2: 2.06 (0.56) Group 3: 4.03 (0.69)
Gagnon 1997 [36], Canada	RCT	Parity 0 to 4, normal pregnancy, ability to speak English, French or Spanish, telephone availability, residence within 30 min of hospital	Discharge between 6 to 36 h, nursing care by telephone within 48 h and at 10 days, home visit at days 3 and 5 postpartum. Prenatal preparation: home visit at 34 and 38 weeks’ gestation (80)	Discharge between 48 to 72 h, and follow-up as determined by woman’s and infant’s physicians (100)	Mean (SD) 29.6 (4.7) vs 29.1 (5.3) Respectively int. and control	Maternal outcomes ▪ Competence in mothering *Perceived Maternal Task Performance Scale* Mean difference (95% CI) at 1 month: 4.3 (−7.7–16.3) Neonatal outcomes ▪ Infant health contacts For feeding, crying, sleeping or care of the umbilical cord RR (95% CI): 0.88 (0.45–1.73) ▪ Infant weight gain Mean difference (95% CI) during the follow-up period 1.2 (−2.8–5.2) ▪ Neonatal hyperbilirubinemia RR (95% CI) 0,50 (0.10–2.50) Breastfeeding (predominantly at 1 month) *Intervention*: 55.1% *Control*: 39.2% *RR (95% CI):* 1.41 (1.02–1.94), ns when adjustment in those to breast-feed at baseline Length of stay: mean in hours (SD) *Intervention*: 37.5 (19.7) *Control:* 54.3 (18.0)
Hellman 1962 [30], USA	RCT	NR	Discharge within 48 h or between 49 and 72 h + midwife home visits within 48 h of discharge, within first week and sometime at 3 weeks (1941)	Discharge after 5 days + midwife home visits within first week and sometime at 3 weeks (316)	Mean 23.6 vs 23.8 Respectively int. and control	Maternal outcomes ▪ Re-admission at 3 weeks Intervention 1.8% Control 0.6%, ns ▪ Complication rate Significant difference in febrility, lochia, involution of the uterus and breast engorgement in favour of control group ▪ Need for advice rate Significant difference between groups *For mother* Intervention 72.6% vs control 63.0% *For newborn* Intervention 72.5% vs control 62.8% Neonatal outcomes ▪ Re-admission rate at 3 weeks Intervention 1.1% vs control 0.6%, ns ▪ Mortality Intervention 0.46% (aspiration of milk, massive bronchopneumonia, unknown reason) vs control 0.24% (heart-disease), ns ▪ Weight gain Results reported in graph (no significant difference) ▪ Complication rate (Morbidity) Intervention 35.1% Control 32.1%, ns Breastfeeding ▪ Rate at 3 weeks Intervention 15% control 6% ▪ Breast engorgement Results reported in graph
Sainz-Bueno 2005 [31], Spain	RCT	Healthy term neonates (34/42 weeks), weigh >2500 g, vaginal delivery with normal evolution	Discharge in the first 24 h with qualified nurse visits over the next 24 to 48 h, monitored at 7 to 10 days in practice, telephone consultation at 1, 3 and 6 months.No prenatal preparation (213)	Discharge after minimum 48 h, monitored at 7 to 10 days in practice, telephone consultation at 1, 3 and 6 months (217)	≤ 30 y. 53.9%	Maternal outcomes ▪ Readmission within 6 weeks RR (95% CI) = 0.81 (0.21–3.03) ▪ Maternal consultation RR (95% CI) = 0.78 (0.30–2.10) ▪ Morbidity RR (95% CI) = 0.73 (0.30–2.20) ▪ Depression *At 1 week* RR (95% CI) = 0.64 (0.25–1.63) *At 1 month* RR (95% CI) = 0.30 (0.33–3.20) ▪ Fatigue *At 1 week* RR (95% CI) = 0.85 (0.43–1.64) *At 1 month* RR (95% CI) = 0.50 (0.04–5.54) Neonatal outcomes readmission within 28 days RR (95% CI) = 0.61 (0.15–2.56) Breastfeeding *At 1 week* RR (95% CI) = 0.48 (0.14–1.65) *At 1 month* RR (95% CI) = 0.58 (0.25–1.36) *At 3 months* RR (95% CI) = 0.62 (0.42–0.91), *p* = 0.16 *At 6 months* RR (95% CI) = 0.68 (0.46–1.0006) *At > 9 months* RR (95% CI) = 0.81 (0.49–1.32) Evaluation of costs Saving of 18.1% by an early discharge program with 1 home visit and telephone follow-up
Smith-Hanrahan 1995 [34], Canada	RCT	English or French speaking, social support at least 12 h/days during 2 frist days, no major obstetrical complications, no prolonged mother-infant separation in hospital, healthy newborn infant with a wiegth between 2500 and 4500 g. feeding established.	Length of stay <60 h, nursing follow-up by phone call starting within 24 h after discharge with home visit in case of need, phone number available 24 h/24 and 7d/7, paediatric and obtretric office visits (35)	Length of stay ≥60 h, paediatric and obtretric office visits (46)	Mean (SD) 29.5 (4.5) vs 29.3 (4.6) Respectively int. and control	Maternal outcomes ▪ Re-admission at 6 weeks Intervention 0/35 Control 0/46, ns Neonatal outcomes ▪ Re-admission at 6 weeks Intervention 0/35 Control 0/46, ns Breastfeeding ▪ Proportion of breastfed neonates at 6 weeks Intervention 17/35 Control 29/46, ns
Waldenström 1987 [35], Sweden	RCT	No significant complication during pregnancy and birth, no significant morbidity in first 24 h for both mother and newborn, gestational age > 37 weeks, birthweight ≥3000 g, Apgar score at 5 min ≥ 7	Discharge between 24 and 48 h, prenatal nurse home visit 4 weeks before term, daily nurse home visit for 3 to 4 days post discharge, paediatric office visit on day 5	Discharge >48 h, standard hospital care without home visit after discharge	Early discharge 28 years vs late discharge 27 years	Maternal outcomes ▪ Re-admission at 6 weeks Intervention 0/50 Control 1/54, ns ▪ Depressed mood in first 6 weeks Intervention 3/50 Control 4/54, ns Neonatal outcomes ▪ Re-admission at 8 weeks Intervention 1/50 Control 0/54, ns Breastfeeding ▪ Proportion of breastfed neonates at 2 months Intervention 37/49 Control 45/52, ns ▪ Proportion of breastfed neonates at 6 months Intervention 28/49 Control 20/52, ns
Yanover 1976 [32], USA	RCT	Parity 0 or 1, 19y < mother age < 35 y low medical risk, ≥ 12th-grade education, father’s attending prenatal preparation, communicate well in English, parents residing together within 32 km of hospital.	Antenatal preparation for child birth, early discharge, father participation. Infant in nursery for 6 h, Release from hospital as soon as the mother and infant (if fulfilled discharge criteria at 12 h). Daily home visits until day 4 postpartum (44)	Antenatal preparation for child birth. Infant in nursery for 24 h, discharge ≥48 h, paediatric visit at 2 weeks, obstetric visit at 6 weeks (44)	NR	Maternal outcomes ▪ Morbidity No difference in frequency and types of morbidity within the first 6 weeks (obstetrical laceration, post-partum infection, postpartum haemorrhage) ▪ Re-admission at 6 weeks Intervention 0% Control 0%, ns Neonatal outcomes ▪ Morbidity No difference in frequency and types of morbidity (need for hospital care: in each group 2 newborns for jaundice, 2 newborn in control group for pneumonia) Evaluation of costs Saving about 30% of daily costs for early discharge Length of stay: median in hours (range) Early discharge: 26 (12–86) Conventional stay: 68 (31–167)

### Definition of early hospital discharge

No standard definition of ‘early discharge’ from hospital was found across trials. A variation in the discharge time from less than 24 h to 72 h was seen. Similarly, the ‘usual length of stay’ ranged from 48 h to 5 days. In the five included trials, all early discharge programs proposed post-discharge home visits by nurses or midwives. However, prenatal preparation for early discharge was not always foreseen. In the two included non-randomised studies, early discharge was defined as a length of stay of maximum 24 h. While one study only proposed a medical visit 7 days after delivery to assess newborn health status, the other study organised two or three home visits, during the first week, performed by two nurse-midwives with experience in neonatal care.

### Early discharge and maternal health outcomes

#### Morbidity

Four trials reported data on maternal morbidity [29–32]. Among these, three studies did not find any difference in complication rates between early discharge and conventional length of stay [29, 31, 32]. The most frequently reported pathologies were urinary tract infection, episiotomy infection, mastitis and other mammary pathologies and endometritis. In contrast, Hellman et al. found a small significant difference in favour of conventional length of stay in comparison with early discharge in febrility, lochia, involution of the uterus, and breast engorgement at 3 weeks of puerperium [30]. No pooling estimation of the rate of complications was done because of the poor quality of data reporting and the large differences in the time frame of morbidity rate measurement.

#### Complication rate

A non-randomised study, performed in a general hospital in Mexico, found no significant difference, during the first week postpartum, between reported complication rate by the mothers discharged at 24 h or less and those discharged later (OR (95% CI) = 0.95 (0.41–2.20). In this study, the prenatal care was assessed according to the Official Mexican Norm (NOM-007-SSA2–1993) [33]. The authors found that women with an early discharge and satisfactory prenatal care had a 64% lower odds for presenting symptoms within the first week postpartum compared with women with being discharged later and from whom prenatal control was unsatisfactory (OR 0.36; 95% CI: 0.17–0.76) [27].

#### Counseling

Hellman et al. showed that mothers discharged early were statistically significantly more likely to require more advice during the follow-up period for themselves or for their newborn in comparison with conventional length of stay mothers [30] (see Table 1). Information requested about newborns related to feeding, bowels, hygiene and care of the umbilical cord whereas breast care, perineal care, personal hygiene and exercise were the most reported topics for advice for mothers.

#### Readmission

**Fig. 2:**
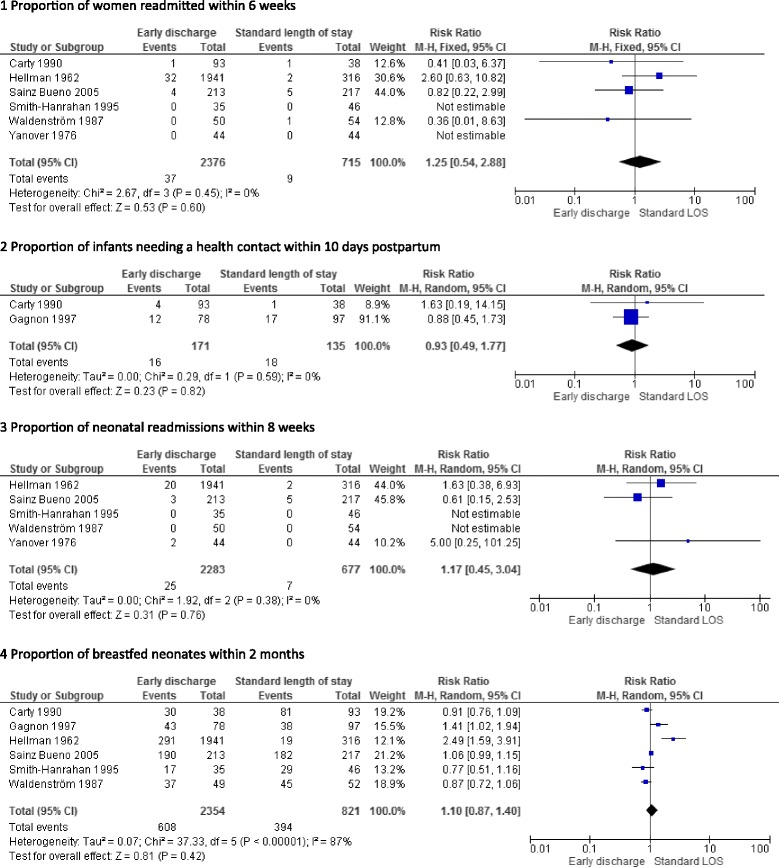
Pooled estimation for maternal and neonate health outcomes

No significant differences in maternal readmission rates during the postpartum period were found in four trials [29–32, 34, 35]. Figure 2 presents a pooled risk ratio (95% confidence interval) (RR (95%CI) of 1.25 (0.54–2.88) for maternal readmissions occurring from week 1 to 6 after birth.

#### Psychological functioning

- Depression

Carty and Bradley found a significant difference in mean scores on the Beck Depression Index (*p* < 0.05) between mothers with length of stay ≤24 h and those with a four-day stay (see Table 1). Mothers with a shorter postpartum stay had better emotional well-being at 1 month than those with a standard duration hospital stay. However, no difference was found between a length of stay of 25 to 48 h and one of 4 days [29].

Sainz-Bueno et al. showed no difference in anxiety and depression between early discharged mothers and those with conventional duration stays measured by the hospital anxiety and depression (HAD) scale [31]. Nevertheless, it should be noted that 2.8% of the mothers had scores on HAD scale at 1 week postpartum indicating a requirement of psychiatric monitoring. Among those, 0.5% required psychiatric admission. At 1 month post-delivery, this proportion reached 2.3%. Nevertheless, no difference was found between early discharge and conventional stay mothers.

Waldenström et al. found no difference in the proportions of women reporting a depressed mood during the first 6 weeks after birth between early discharge (26%) and conventional stay (34%) [35]. Reported depressed mood peaked during the 2nd and 3rd week after the birth.

Because of existing differences in the scales used for measuring depression, no pooling of data was performed.

- Competence of mothering

Gagnon et al. measured competence in mothering using the Perceived Maternal Task Performance Scale at 1 month [36]. Competence in mothering included mothers’ ability to assess their infant’s needs and their own abilities to feed their newborn and to perform caregiving activities. The authors found no significant difference between mean scores between mothers discharged between 6 h and 36 h post-delivery and those discharged between 42 to 72 h.

Another randomised trial assessed women’s confidence in their mothering skills using an eight-item scale [29]. The authors reported a significantly higher score at 1 week in mothers discharged within 24 h than those discharged 4 days post-delivery (see Table 1). After a month, this difference did not remain. In addition, no differences were noted between very early discharged mothers (between 12 and 24 h) and early discharged mothers (between 25 and 48 h).

- Sense of security

Askelsdottir et al. reported a greater sense of security in the first postnatal week for early discharged women [16]. However, those women had more negative emotions towards breastfeeding compared with women with conventional length of stay. Because there is no difference in parity between the 2 groups, an explanation can be found in a higher educational level of mothers included in the group with conventional length of stay. Contact between the mother, newborn and partner did not differ between the two lengths of stay.

### Early discharge and neonate health outcomes

#### Mortality

Only one trial assessed the mortality rate in newborns at 3 weeks of life [30]. The authors found mortality rates of 0.24% and 0.46% in newborns with early discharged conventional babies and length of stay respectively. These proportions were not statistically different.

#### Morbidity

Two trials reported the proportion of newborns needing a health contact within 10 days postpartum [29, 36]. Pooling of results does not show any difference between early discharged newborns and those who stayed longer in hospital after birth (see Fig. 2).

Yanover et al. concluded that no significant differences in type or in occurrence of neonatal morbidities were observed during the follow-up period [32]. Unfortunately, no data were reported. After 3 weeks, Hellman et al. did not find any significant difference in complication rates between early discharged newborns and those with conventional duration stays [30] (see Table 1).

#### Weight gain

Two trials analysed the difference in weight gain of newborns with early discharge and conventional length of stay [30, 36]. The first study did not find any significant differences in daily rates of infant weight gain during the first 10 days of life [36] (see Table 1). The second trial focused on weight gains at 3 weeks of life [30]. No significant difference was found between early discharged newborns and infants with a standard length of stay.

#### Neonatal hyperbilirubinemia

One trial studied the occurrence of hyperbilirubinemia [36] and reported that there was no difference in identification of significant neonatal hyperbilirubinemia (see Table 1). However, a significantly reduced crude relative risk for bilirubin testing (RR [CI 95%]: 0.39 [0.17–0.94]) was observed in early discharged neonates. Notwithstanding, the proportion of tested newborns eligible for phototherapy were not significantly different (RR [CI 95%]: 1.26 [0.33–4.93]).

#### Readmission

No significant differences in neonatal readmission rates were found in the three trials reporting data on this outcome [30–32, 34, 35]. The pooled RR for neonatal readmissions occurring within 3 to 8 weeks of birth is presented in Fig. 2 and does not show any increased risk of readmission in early discharged neonates.

### Breastfeeding

Six trials reported data on partial or exclusive breastfeeding from 3 weeks to 9 months. Pooled estimates are presented in Fig. 2 and show that early discharge mothers were equally likely to breastfeed their neonates than those with a conventional length of stay. Waldenström et al. found no difference in breastfeeding rate at 6 months according to the length of stay [35]. Sainz-Bueno et al. studied breastfeeding rates up to 9 months and did not find any differences between the groups at any time except at 3 months with a significantly higher rate in early discharged mothers (see Table 1).

A non-randomised study reported that early discharge mothers (between 12 to 24 h postpartum) described less positive emotions towards breastfeeding compared with other mothers (conventional length of stay from 24 to 48 h postpartum) [16]. Emotions towards breastfeeding were measured using the 2 domains of the Alliance scale (Alliance scale mean score (SD) Breastfeeding strain: early discharge: 2.4 (1.31), conventional length of stay: 1.7 (0.93); *p* = 0.001/Breastfeeding uncomfortable early discharge: 2.9 (1.61), conventional length of stay: 2.2 (1.07); *p* = 0.028).

### Quality assessment of included studies and level of evidence

The only systematic review included [5] was rated at a level of 9/11 with the AMSTAR check list (see Additional file 1). Despite its high quality, the review was only used as a source of evidence because the research questions were slightly different with our review allowing the inclusion of caesarean sections within its scope. The quality appraisal of all RCTs matching our inclusion criteria is shown in Additional file 1. The lack of blinding of participants and personnel was not feasible and was not considered a risk of bias in the context of postpartum care because it does not affect the outcomes assessed. Methods used to ensure allocation concealment were reported in three trials [29, 31, 36] while the remaining two did not provided information on this methodological item. None of the included studies indicated if outcomes assessment was blinded. In addition to the specific bias linked to their non-randomised design, the two comparative studies presented other bias such as single centre design, selection biases or small sample size. Details on quality assessment of these studies are reported in Additional file 1.

The level of evidence is documented in Additional file 1. Despite RCT design, the vast majority of outcomes are rated as low or very low level of evidence. Outcomes regarding counselling, neonatal mortality rate and rate of health contact for newborns within 10 day of life are rated as moderate.

## Discussion

Based on the available evidence, early postpartum discharge seems to be clinically safe for both mothers and newborns. These results are in line with a previously published review [5] which was restricted to RCTs but included caesarean sections. Early hospital discharge in caesarean section deliveries did not seem to affect outcome such as breastfeeding rate, maternal and infant readmission. However, confounders such as type of health system (public versus private), opportunities for antenatal preparation and for postnatal domiciliary midwife support or type of postpartum intervention cannot be studied because of poor reporting. Therefore, mixing data from vaginal delivery and caesarean section must considered with caution. Despite of this precaution, evidence must be interpreted with caution for two main reasons.

Firstly, the **concept of early hospital discharge** and that of conventional length of stay varied greatly between trials, making an interpretation or comparison across studies difficult. Among the selected studies, a discharge was considered early when the mother-infant dyad was discharged from 6 h to 72 h after birth. Currently, there are considerable variations in length of stay between Western countries. In Sweden, early discharge corresponds to a length of stay of 6 h after birth [3, 37], while in France, a discharge after a length of stay of less than 72 h is considered as early [19]. In the US, a minimum period of 48 h is ensured by law after a resurgence of jaundice cases and an increase in re-admissions among both mothers and children [1, 2]. However, Evan et al. [1] demonstrated that the extension of the postpartum length of stay to 48 h had little impact on re-admission rates for vaginally delivered newborns and was not cost saving. A lack of home postpartum follow-up may explain the absence of saving. Many Western countries already practiced early postpartum discharge for more than a decade. For these countries, what was once considered early discharge is now a conventional length of postnatal stay. This may explain the lack of recent published RCTs on the subject. However, countries with longer lengths of stay such as France and Belgium may benefit from testing shorter lengths of stay in studies with an appropriate design.

The second reason to interpret the findings with caution relates to the **quality of the included studies**. GRADE assessment highlighted numerous limitations in methods leading to (very) low level of evidence (see Additional file 1), except for three outcomes with moderate level of evidence (counselling, neonatal mortality rate at 3 weeks, and need for neonatal health contact within 10 days). The outcome related to satisfaction, only reported in the RCTs, was not discussed in this systematic review because trials were performed from 10 to 39 years ago, and thus societal expectations on health care are likely to have dramatically changed. Among the main methodological limitations found in the included studies were: small sample sizes that lead to insufficient power, a large number of lost to follow-up, one study with poor reporting of outcomes using graphical presentation but no discussion on the data in numerical terms, and heterogeneity in results across studies, leading to conflicting conclusions.

In addition, the selection criteria of study participants in RCTs were too restrictive, including well-educated women, mothers living in a stable relationship with their partners and low socioeconomic risk, preventing the generalisation of findings to the real world population. Selection bias was also observed in one non-randomised study [27] because more healthy mothers and newborns without financial difficulties were included in the early discharged group.

Besides the cautions in evidence interpretation, approximating the number of mother-infant dyads that can benefit from early discharge is challenging. Yanover et al. estimated that 25% of all deliveries are eligible for early discharge (i.e. after approximately 1 day). The authors used the readiness of the family as a necessary condition for discharge [32]. This criterion is indeed crucial for a successful early discharge programme because of a positive association between unreadiness at postpartum discharge and, on the one hand, health care use and, on the other hand, poorer health outcomes within 4 weeks after discharge [18]. The readiness to be discharged can be defined as the agreement between the mother and the medical staff (i.e. paediatrician and obstetrician) that both mother and newborn will not be likely to benefit from a longer hospital stay. If one of the involved parties anticipates any potential benefits for mother or infant to stay longer, the dyad is considered as unready [18].

Therefore, early discharge criteria cannot only focus on clinical parameters as described in Sainz et al. [31] but should also include additional criteria regarding the prenatal preparation and the setting of home postpartum follow-up as foreseen in the French guideline on early postpartum discharge [38]. In addition, prenatal preparation, content, frequency and duration of home follow-up were extremely different across studies hampering our ability to highlight best practices in these domains. In a Cochrane systematic review, the authors assessed the outcomes for women and babies of different home-visit schedules up to 42 days after birth whatever the length of hospital stay [39]. They concluded that postnatal home visits may promote infant health and maternal satisfaction but the frequency, timing and duration of such postnatal care visits should be based upon local needs. As in our systematic review, an optimal package of home postnatal care cannot be defined. However, several countries are developing a range of strategies to deliver seamless individualized follow-up support using telemedicine tools such as video conferencing or apps [40]. Nevertheless, all those strategies are unlikely to respond fully to the existing concerns about continuity of care. In addition, the shift of the follow-up tasks from hospital to home with physically or virtually present carers raises concerns about medical responsibility and legal accountability. Finally, the availability of professionals to provide the home care is an essential prerequisite for the successful transition from hospital to postpartum home care without impairing the home care for other conditions [41], creating unmet needs, or using informal support workers as doulas [42]. Due to the risk of variations in content and quality of care provided by fragmented services across multiple carers, offering a seamless transition from hospital to home is challenging.

Cost containment is one of the drivers to endorse an early postpartum discharge policy. Only two trials included in our systematic review addressed the cost issue. In the first study implemented in the USA [32],the authors analysed an early discharge programme, which included 24 h hospital length of stay and daily home visits by trained nurses for 4 days after birth. From a hospital perspective, this programme provided a minimum of 30% immediate savings in daily costs versus conventional stay (48 h or more), excluding saving generated by allocating vacated beds and rooms to other patients or purposes. The cost calculation was based on expenses for salaries of nurse practitioners, paramedical personnel, and medical consultants, as well as automobile expenses and home-care supplies. In the second study performing a cost calculation from a payer perspective in Spain [31], the implementation of an early discharge programme provided savings ranging from 18 to 20% in comparison with convention hospital length of hospital stay. This early discharge programme was made up of a less than 48 h length of hospital stay, 1 home visit after discharge by nurses qualified in puerperal and neonatal care, a practice consultation between 7 to 10 days after birth and a follow-up by phone at 1, 3 and 6 months. By comparison, the conventional discharge programme followed the same pattern except for the length of stay, which was longer than 48 h and the absence of home visits. The cost calculation took into account costs related to length of hospital stay, maternal – neonatal readmission, maternal – neonatal consultations, maternal – neonatal reassessment at 1 week and costs related to telephone follow-up. Cost containment due to the implementation of an early discharge policy is highly dependent on the perspective taken into account as well as on the type of health insurance system in place.

Estimation of possible cost savings cannot be performed without developing well designed RCTs including costs data collection. The collection of cost data must be designed to take into account not only hospital or payer perspectives, but also the societal perspective. In addition, the hidden costs for new mothers created by a potential shift from publicly funded hospital maternity care to private home-based postpartum care must be carefully scrutinized to prevent gaps in quality and accessibility of care [42].

Finally, developing quality indicators to monitor each early postpartum discharge programme is also crucial. Quality indicator assessment allows for adjusting early postpartum discharge policy when needed. However, the development of indicators is challenging because some indicators (e.g. satisfaction, overall support, well-being …) are qualitative and, thus more difficult to standardize [43].

## Conclusions

The current available literature provides little scientific evidence to guide postpartum discharge policy planning. The evidence based on RCTs is old, with the most recent trial published 10 years ago, and the quality of evidence of these trials is poor. The more recent evidence is based only on two very poor quality non-randomised studies. In addition, the concept of early discharge itself is very variable across studies leading to health outcomes being measured at variable times after delivery. Despite these limitations, early discharge seems to be safe for both mother and newborn. Breastfeeding did not seem to be affected. Because of the lack of robust clinical evidence and full economic evaluations, the current data neither support nor discourage the widespread use of early postpartum discharge. Before implementing an early discharge policy, Western countries with longer length of hospital stay such as France and Belgium may benefit from testing shorter length of stay in studies with an appropriate design (e.g. randomised). The issue of cost containment in implementing early discharge and the potential impact on the current and future health of the population exemplifies the need for publicly funded clinical trials in such public health area. Finally, trials testing the range of out-patient interventions supporting early discharge are needed in Western countries which implemented early discharge policies in the past.

## Additional file

Additional file 1: Additional materials report detailed search strategy by database, assessment of the evidence using AMSTAR and Cochrane risk of bias tools, list of excluded studies including full references and level of evidence using GRADE system. (DOCX 125 kb)

## Abbreviations

CI: Confidence interval
HAD scale: Hospital anxiety and depression scale
OR: Odd ratio
RR: Risk ratio
