# Supplementary material for: Vaginal delivery: how does early hospital discharge affect mother and child outcomes? A systematic literature review
Source: BMC Pregnancy Childbirth. 2017 Sep 6;17:289. doi: 10.1186/s12884-017-1465-7 (PMC5588709; doi:10.1186/s12884-017-1465-7)
Supplement: Additional file 1: — Additional materials report detailed search strategy by database, assessment of the evidence using AMSTAR and Cochrane risk of bias tools, list of excluded studies including full references and level of evidence using GRADE system. (DOCX 125 kb) [file 12884_2017_1465_MOESM1_ESM.docx]

Vaginal delivery: how does early discharge affect mother and child outcomes? A literature review: additional material

Table of Contents

[1 Additional material 1: search strategy 2](#_Toc436649159)

[1.1 Cochrane Database of Systematic Reviews 2](#_Toc436649160)

[1.2 CINAHL 3](#_Toc436649161)

[1.3 Econlit <1886 to July 2015> 5](#_Toc436649162)

[1.4 Embase 6](#_Toc436649163)

[1.5 Medline OvidSP 7](#_Toc436649164)

[2 Additional material 2: methodological quality assessment of the evidence 9](#_Toc436649165)

[3 Additional material 3: list of excluded studies 11](#_Toc436649166)

[4 Additional material 4: Grading of evidence 13](#_Toc436649167)

[5 References 18](#_Toc436649168)

# Additional material 1: search strategy

## Cochrane Database of Systematic Reviews

| **Date** | 03/06/15 16:33:30.967 | |
| --- | --- | --- |
| **Database** | Cochrane Database of Systematic Reviews | |
| **Search strategy** | | |
| #1 | MeSH descriptor: [Infant, Newborn] explode all trees | 13385 |
| #2 | MeSH descriptor: [Mothers] explode all trees | 1017 |
| #3 | MeSH descriptor: [Midwifery] explode all trees | 262 |
| #4 | MeSH descriptor: [Postpartum Period] explode all trees | 1099 |
| #5 | MeSH descriptor: [Maternal Health Services] explode all trees | 1651 |
| #6 | MeSH descriptor: [Maternal Welfare] explode all trees | 140 |
| #7 | (newborn or newborns):ab,ti | 4669 |
| #8 | (neonate or neonates):ab,ti | 4134 |
| #9 | postpartum:ab,ti | 3242 |
| #10 | puerperium:ab,ti | 143 |
| #11 | (mother or mothers):ab,ti | 7086 |
| #12 | (postnatal or post-natal):ab,ti | 1838 |
| #13 | #1 or #2 or #3 or #4 or #5 or #6 or #7 or #8 or #9 or #10 or #11 or #12 | 25707 |
| #14 | MeSH descriptor: [Length of Stay] explode all trees | 6801 |
| #15 | MeSH descriptor: [Patient Discharge] explode all trees | 1119 |
| #16 | "length of stay":ab,ti | 3262 |
| #17 | discharg*:ab,ti | 12980 |
| #18 | (day or days):ti | 6520 |
| #19 | stay*:ti | 702 |
| #20 | #14 or #15 or #16 or #17 or #18 or #19 | 26772 |
| #21 | #13 and #20 | 1563 |
| #22 | MeSH descriptor: [Premature Birth] explode all trees | 420 |
| #23 | MeSH descriptor: [Retinopathy of Prematurity] explode all trees | 252 |
| #24 | MeSH descriptor: [Infant, Premature, Diseases] explode all trees | 2228 |
| #25 | MeSH descriptor: [Infant, Premature] explode all trees | 2821 |
| #26 | MeSH descriptor: [Infant, Low Birth Weight] explode all trees | 1837 |
| #27 | (preterm or pretarms or prematur*):ti | 7628 |
| #28 | "low birth weight":ti | 1456 |
| #29 | #22 or #23 or #24 or #25 or #26 or #27 or #28 | 10663 |
| #30 | #21 not #29 | 855 |
| #31 | #21 not #29 Publication Year from 2008 to 2015 | 369 |
| Notes | The search has been updated on 25/08/2015 resulting in 4 more results. The search was performed on Wiley interface which allows to search several databases. Here is the detail of the hits found in each database:  Cochrane Database of Systematic review 39  Cochrane CENTRAL 302  HTA database 2  CRD economic evaluation 21  DARE 8  Cochrane methodology register 1  The latest database was not used for this review. | |

## CINAHL

| **Date** | 2015-06-04 | |
| --- | --- | --- |
| **Database** | CINAHL | |
| **Search strategy** | | |
| S1 | MH Infant, Newborn | 87,231 |
| S2 | MH Mothers | 17,796 |
| S3 | MH Midwifery/ | 13,698 |
| S4 | MH Postpartum period | 0 |
| S5 | MW postpartum | 5,615 |
| S6 | MH Maternal Health Services | 5,495 |
| S7 | MW puerperium | 577 |
| S8 | MH maternal welfare | 1,122 |
| S9 | (MH "Postpartum Care (Saba CCC)") | 2 |
| S10 | (MH "Postnatal Care+") | 3,414 |
| S11 | TI postnatal OR AB postnatal | 6,734 |
| S12 | TI post-natal OR AB post-natal | 398 |
| S13 | TI newborn# OR AB newborn# | 15,050 |
| S14 | TI neonate# OR AB neonate# | 10,147 |
| S15 | TI postpartum OR AB postpartum | 9,829 |
| S16 | TI puerperium OR AB puerperium | 401 |
| S17 | TI mother# OR AB mother# | 36,896 |
| S18 | (S1 OR S2 OR S3 OR S4 OR S5 OR S6 OR S7 OR S8 OR S9 OR S10 OR S11 OR S12 OR S13 OR S14 OR S15 OR S16 OR S17) | 156,608 |
| S19 | (MH "Length of Stay") | 24,115 |
| S20 | (MH "Patient discharge") | 10,555 |
| S21 | TI "length of stay" OR AB "length of stay" | 9,529 |
| S22 | TI discharg* OR AB discharg* | 33,452 |
| S23 | TI day# | 18,908 |
| S24 | TI stay* | 7,755 |
| S25 | (MH "Early Patient Discharge") | 753 |
| S26 | (MH "Discharge Planning") | 3,800 |
| S27 | (MH "Discharge Planning") | 3,800 |
| S28 | (MH "Patient Discharge Education") | 1,520 |
| S29 | (MH "Bed Occupancy") | 2,832 |
| S30 | (S19 OR S20 OR S21 OR S22 OR S23 OR S24 OR S25 OR S26 OR S27 OR S28 OR S29) | 88,383 |
| S31 | S18 AND S30 | 6,203 |
| S32 | S18 AND S30 | 3,112 |
| S33 | MH premature birth | 0 |
| S34 | MH "Retinopathy of Prematurity" | 971 |
| S35 | MH Infant, Premature, Diseases | 2,733 |
| S36 | MH Infant, Premature | 14,840 |
| S37 | MH Infant, Low Birth Weight+ | 9,020 |
| S38 | TI (preterm# or prematur*) | 15,359 |
| S39 | S33 OR S34 OR S35 OR S36 OR S37 OR S38 | 28,711 |
| S40 | S32 NOT S39 | 2,299 |
| S41 | S32 NOT S39 | 567 |
| S42 | (TX MEDLINE) OR (TX "systematic review") OR (MH "systematic review+") OR (MH "meta analysis") OR (TI intervention*) | 60,816 |
| S43 | S41 AND S42 | 60 |
| S44 | (MH "Clinical Trials+") | 192,248 |
| S45 | PT Clinical trial | 78,692 |
| S46 | TX clinic* n1 trial* | 252,765 |
| S47 | TX ( (singl* n1 blind*) or (singl* n1 mask*) ) or TX ( (doubl* n1 blind*) or (doubl* n1 mask*) ) or TX ( (tripl* n1 blind*) or (tripl* n1 mask*) ) or TX ( (trebl* n1 blind*) or (trebl* n1 mask*) ) | 803,250 |
| S48 | TX randomi* control* trial* | 157,210 |
| S49 | (MH "Random Assignment") | 39,835 |
| S50 | TX random* allocat* | 12,273 |
| S51 | TX placebo* | 79,971 |
| S52 | (MH "Placebos") | 9,388 |
| S53 | (MH "Quantitative Studies") | 13,659 |
| S54 | TX allocat* random* | 12,273 |
| S55 | S44 OR S45 OR S46 OR S47 OR S48 OR S49 OR S50 OR S51 OR S52 OR S53 OR S54 | 1,073,510 |
| S56 | S41 AND S55 | 216 |
| S57 | TX (early n3 discharge) | 4,953 |
| S58 | S18 AND S57 | 135 |
| S59 | S58 NOT (S56 OR S43) | 124 |
| S60 | S59 NOT S39 | 87 |
| Notes | Results exported from lines S43, S56 and S60. An update has been performed on 25/08/2015 resulting in a total of 322 references (5 more). | |

## Econlit <1886 to July 2015>

| **Date** | 2015-08-25 | |
| --- | --- | --- |
| **Database** | Econlit <1886 to July 2015> | |
| **Search strategy** | | |
| 1 | newborn?.mp. | 219 |
| 2 | midwifery.mp. | 19 |
| 3 | postpartum.mp. | 64 |
| 4 | "maternal health service".mp. | 5 |
| 5 | "maternal welfare".ab,ti. | 3 |
| 6 | puerperium.mp. | 0 |
| 7 | neonate?.mp. | 8 |
| 8 | mother?.mp. | 3676 |
| 9 | post?natal.mp. | 32 |
| 10 | 1 or 2 or 3 or 4 or 5 or 6 or 7 or 8 or 9 | 3922 |
| 11 | "length of stay".mp. | 317 |
| 12 | "patient discharge".mp. | 11 |
| 13 | discharg*.mp. | 920 |
| 14 | day?.mp. | 11153 |
| 15 | stay*.mp. | 3604 |
| 16 | 11 or 12 or 13 or 14 or 15 | 15375 |
| 17 | 10 and 16 | 228 |
| 18 | limit 17 to yr="2008 -Current" | 129 |
| Notes |  | |

## Embase

| **Date** | 2015-06-03 | |
| --- | --- | --- |
| **Database** | Embase | |
| **Search strategy** | | |
| 1 | 'newborn'/exp | 489001 |
| 2 | 'newborn':ab,ti | 124104 |
| 3 | 'mother'/exp | 97486 |
| 4 | 'mother':ab,ti OR mothers:ab,ti | 195478 |
| 5 | 'midwife'/exp | 25082 |
| 6 | 'puerperium'/exp | 46608 |
| 7 | 'puerperium':ab,ti | 6291 |
| 8 | postpartum:ab,ti | 44005 |
| 9 | neonate*:ab,ti | 86721 |
| 10 | 'maternal care'/exp | 32201 |
| 11 | 'maternal welfare'/exp | 11125 |
| 12 | #1 OR #2 OR #3 OR #4 OR #5 OR #6 OR #7 OR #8 OR #9 OR #10 OR #11 | 826350 |
| 13 | 'length of stay'/exp | 98176 |
| 14 | 'length of stay':ab,ti | 52229 |
| 15 | 'hospital discharge'/exp | 71091 |
| 16 | discharg*:ab,ti | 262462 |
| 17 | day:ti OR days:ti | 59016 |
| 18 | stay*:ti | 13443 |
| 19 | #13 OR #14 OR #15 OR #16 OR #17 OR #18 | 432160 |
| 20 | #12 AND #19 | 23725 |
| 21 | 'prematurity'/exp | 78271 |
| 22 | 'premature mortality'/exp | 909 |
| 23 | prematur*:ti | 38453 |
| 24 | preterm:ti OR preterms:ti | 28216 |
| 25 | 'low birth weight'/exp | 42918 |
| 26 | 'low birth weight':ti | 9079 |
| 27 | #21 OR #22 OR #23 OR #24 OR #25 OR #26 | 135724 |
| 28 | #20 NOT #27 | 19266 |
| 29 | #20 NOT #27 AND [2008-2015]/py | 9158 |
| 30 | #29 NOT [medline]/lim | 4604 |
| 31 | 'meta-analysis'/exp OR 'meta-analysis' OR 'systematic review'/exp OR 'systematic review' | 205019 |
| 32 | #30 AND #31 | 41 |
| 33 | random*:ab,ti OR placebo*:de,ab,ti OR (double NEXT/1 blind*):ab,ti | 1202703 |
| 34 | #30 AND #33 | 292 |
| 35 | (early NEAR/3 discharge):ab,ti | 4337 |
| 36 | #12 AND #35 | 676 |
| 37 | #36 NOT [medline]/lim | 118 |
| 38 | #36 NOT [medline]/lim AND [2008-2015]/py | 78 |
| Notes | Results exported from lines 32, 34 and 38. An update has been performed on 25/08/2015 resulting in a total of 417 references (36 more). | |

## Medline OvidSP

| **Date** | 2015-06-04 | |
| --- | --- | --- |
| **Database** | Medline OvidSP | |
| **Search strategy** | | |
| 1 | Infant, Newborn/ | 505037 |
| 2 | Mothers/ | 27712 |
| 3 | Midwifery/ | 15284 |
| 4 | Postpartum period/ | 18117 |
| 5 | Maternal Health Service/ | 10639 |
| 6 | Maternal welfare/ | 6203 |
| 7 | newborn?.ab,ti. | 131865 |
| 8 | neonate?.ab,ti. | 68340 |
| 9 | postpartum.ab,ti. | 36650 |
| 10 | puerperium.ab,ti. | 5306 |
| 11 | mother?.ab,ti. | 158842 |
| 12 | post?natal.ab,ti. | 82184 |
| 13 | 1 or 2 or 3 or 4 or 5 or 6 or 7 or 8 or 9 or 10 or 11 or 12 | 799864 |
| 14 | Length of Stay/ | 62386 |
| 15 | Patient discharge/ | 20156 |
| 16 | "length of stay".ab,ti. | 31490 |
| 17 | discharg*.ab,ti. | 180398 |
| 18 | day?.ti. | 47047 |
| 19 | stay*.ti. | 10247 |
| 20 | 14 or 15 or 16 or 17 or 18 or 19 | 297349 |
| 21 | 13 and 20 | 20710 |
| 22 | Premature Birth/ | 7460 |
| 23 | "Retinopathy of Prematurity"/ | 4710 |
| 24 | Infant, Premature, Diseases/ | 18274 |
| 25 | Infant, Premature/ | 42524 |
| 26 | Infant, Low Birth Weight/ | 15653 |
| 27 | (preterm? or prematur*).ti. | 53398 |
| 28 | "low birth weight".ti. | 7715 |
| 29 | 22 or 23 or 24 or 25 or 26 or 27 or 28 | 97054 |
| 30 | 21 not 29 | 16422 |
| 31 | randomized controlled trial.pt. | 395864 |
| 32 | controlled clinical trial.pt. | 89548 |
| 33 | randomized.ti,ab. | 344870 |
| 34 | placebo.ti,ab. | 167349 |
| 35 | clinical trials as topic/ | 173005 |
| 36 | randomly.ti,ab. | 232157 |
| 37 | trial?.ti. | 188697 |
| 38 | 31 or 32 or 33 or 34 or 35 or 36 or 37 | 985927 |
| 39 | exp animal/ not humans/ | 4043807 |
| 40 | 38 not 39 | 909722 |
| 41 | 30 and 40 | 1007 |
| 42 | limit 41 to yr="2008 -Current" | 422 |
| 43 | limit 30 to systematic reviews | 322 |
| 44 | limit 43 to yr="2008 -Current" | 183 |
| 45 | (early adj3 discharge).mp. [mp=title, abstract, original title, name of substance word, subject heading word, keyword heading word, protocol supplementary concept word, rare disease supplementary concept word, unique identifier] | 3001 |
| 46 | 13 and 45 | 656 |
| 47 | limit 46 to yr="2008 -Current" | 129 |
| 48 | 47 not (42 or 44) | 123 |
| 49 | 42 or 44 or 47 | 679 |
| 50 | remove duplicates from 42 | 392 |
| 51 | remove duplicates from 44 | 166 |
| 52 | remove duplicates from 48 | 121 |
| 53 | remove duplicates from 49 | 636 |
| Notes | Results exported from lines 50, 51 and 52. An update has been performed on 25/08/2015 resulting in a total of 653 references (17 more). | |

# Additional material 2: methodological quality assessment of the evidence

Table 1: AMSTAR – methodological quality assessment of Brown’s systematic reviews[1]

| **1. Was an 'a priori' design provided?**  The research question and inclusion criteria should be established before the conduct of the review. | 🗷 Yes   - No - Can’t answer - Not applicable |
| --- | --- |
| **2. Was there duplicate study selection and data extraction?**  There should be at least two independent data extractors and a consensus procedure for disagreements should be in place | 🗷 Yes   - No - Can’t answer - Not applicable |
| **3. Was a comprehensive literature search performed?**  At least two electronic sources should be searched. The report must include years and databases used (e.g. Central, EMBASE, and MEDLINE). Key words and/or MESH terms must be stated and where feasible the search strategy should be provided. All searches should be supplemented by consulting current contents, reviews, textbooks, specialized registers, or experts in the particular field of study, and by reviewing the references in the studies found. | 🗷 Yes   - No - Can’t answer - Not applicable |
| **4. Was the status of publication (i.e. grey literature) used as an inclusion criterion?**  The authors should state that they searched for reports regardless of their publication type. The authors should state whether or not they excluded any reports (from the systematic review), based on their publication status, language etc. | 🗷 Yes   - No - Can’t answer - Not applicable |
| **5. Was a list of studies (included and excluded) provided?**  A list of included and excluded studies should be provided. | 🗷 Yes   - No - Can’t answer - Not applicable |
| **6. Were the characteristics of the included studies provided?**  In an aggregated form such as a table, data from the original studies should be provided on the participants, interventions and outcomes. The ranges of characteristics in all the studies analysed e.g. age, race, sex, relevant socioeconomic data, disease status, duration, severity, or other diseases should be reported. | 🗷 Yes   - No - Can’t answer - Not applicable |
| **7. Was the scientific quality of the included studies assessed and documented?**  'A priori' methods of assessment should be provided (e.g., for effectiveness studies if the author(s) chose to include only randomized, double-blind, placebo controlled studies, or allocation concealment as inclusion criteria); for other types of studies alternative items will be relevant. | 🗷 Yes   - No - Can’t answer - Not applicable |
| **8. Was the scientific quality of the included studies used appropriately in formulating conclusions?**  The results of the methodological rigor and scientific quality should be considered in the analysis and the conclusions of the review, and explicitly stated in formulating recommendations. | 🗷 Yes   - No - Can’t answer - Not applicable |
| **9. Were the methods used to combine the findings of studies appropriate?**  For the pooled results, a test should be done to ensure the studies were combinable, to assess their homogeneity (i.e. Chi squared test for homogeneity, I2). If heterogeneity exists a random effects model should be used and/or the clinical appropriateness of combining should be taken into consideration (i.e. is it sensible to combine?). | 🗷 Yes   - No - Can’t answer - Not applicable |
| **10. Was the likelihood of publication bias assessed?**  An assessment of publication bias should include a combination of graphical aids (e.g., funnel plot, other available tests) and/or statistical tests (e.g., Egger regression test).  *Note: no graphical aids provided or statistical tests* | - Yes   🗷 No   - Can’t answer - Not applicable |
| **11. Was the conflict of interest stated?**  Potential sources of support should be clearly acknowledged in both the systematic review and the included studies.  Note: *yes for the review, no for the included studies* | - Yes   🗷 No   - Can’t answer - Not applicable |

*Source:* [*Shea BJ*](http://www.ncbi.nlm.nih.gov/pubmed/?term=Shea%20BJ%5BAuthor%5D&cauthor=true&cauthor_uid=17302989) *et al. Development of AMSTAR: a measurement tool to assess the methodological quality of systematic reviews.* [*BMC Med Res Methodol.*](http://www.ncbi.nlm.nih.gov/pubmed/17302989) *2007; 7:10.*

Figure 1: Quality assessment of RCTs (the Cochrane risk of bias tool)


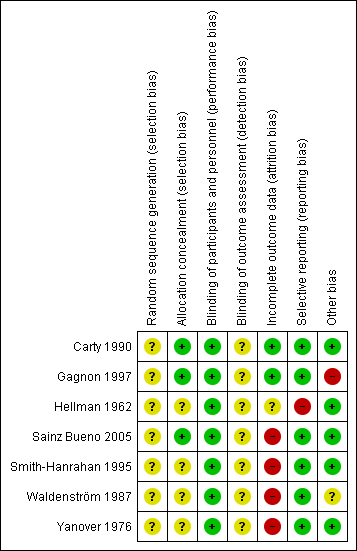


Table 2: Quality assessment of non-randomized studies (the Cochrane risk of bias tool)

|  | Askelsdottir 2013[2] | Ramirez-Villalobos 2009[3] |
| --- | --- | --- |
| 1. Was  selection  of  exposed  and  non‐exposed  cohorts  drawn  from  the  same  population? | Low | Low |
| 2. Can  we  be  confident  in  the  assessment  of  exposure? | Low | Low |
| 3. Can  we  be  confident  that  the  outcome  of  interest  was  not  present  at  start  of  study | Unclear  (Probably no) | Unclear  (Probably no) |
| 4. Did  the  study  match  exposed  and  unexposed  for  all  variables  that  are  associated  with  the  outcome  of  interest  or  did  the  statistical  analysis  adjust  for  these prognostic  variables? | High | Unclear  (Mostly yes) |
| 5. Can  we  be  confident  in  the  assessment  of  the  presence  or  absence  of  prognostic  factors? | Low | Unclear  (Probably yes) |
| 6. Can  we  be  confident  in  the  assessment  of  outcome? | High | High |
| 7. Was  the  follow  up  of  cohorts  adequate? | Unclear  (Probably yes) | High |
| 8. Were  co‐Interventions  similar  between  groups? | Unclear  (Probably yes) | Unclear  (Probably yes) |

# Additional material 3: list of excluded studies

| Almond 2011[4] | Design: not a comparative study |
| --- | --- |
| Barker 2013[5] | Not retrievable |
| Batu 2014[6] | Population: turkey |
| Benitz 2015[7] | Outcomes limited to delivery |
| Bernstein 2013[8] | Population: no comparison between early discharge and longer stay |
| Boulvain 2004[9] | Population: RCT including C-section |
| Bravo 2011[10] | Design: narrative review |
| Brooten 1994[11] | Population: RCT including C-section |
| Cambonie 2010 [12] | Population: Cohort study including C-section |
| Chanot 2009[13] | Population: comparative study including C-section |
| Chanot 2009[14] | Design: not a comparative study |
| Cuncarr 2011[15] | Design: narrative review |
| Das 2014[16] | Intervention |
| De Carolis 2014[17] | Design: not a comparative study |
| De Luca 2009[18] | Design: not a comparative study |
| Evans 2008[19] | Design: not a comparative study |
| Farhat 2011[20] | Population: sample including C-section |
| Fink 2011[21] | Design: not a comparative study |
| Forster 2014[22] | Design: protocol publication |
| Ghedin 2011[23] | Type of publication : poster presentation |
| Gotink 2013[24] | Population: home birth was a comparator |
| Houghton 2008[25] | Design: not a comparative study |
| Lain 2015[26] | Population: Cohort study including C-section |
| McIntosh 2012[27] | Design: not a comparative study |
| Skene 2012[28] | Design: narrative review |
| Soares da Nãbrega Mazzo 2012[29] | Population : early discharge not discussed |
| Straczek 2008[30] | Design: not a comparative study |
| Winterburn 2000[31] | Population: RCT including C-section |
| Yonemoto 2013[32] | Intervention: no inclusion of hospital care in the comparator |

# Additional material 4: Grading of evidence

| Results | No. of studies | 1 | 2 | 3 | 4 | 5 | Reasons for downgrading | GRADE |
| --- | --- | --- | --- | --- | --- | --- | --- | --- |
| **Maternal health outcomes** | | | | | | | | |
| **Morbidity rate**  Carty 1990, Hellman 1962, Sainz-Bueno 2005, Yanover 1976[33-36] | 4 RCT | -1 | -1 | 0 | 0 | -2 | 1. Two trials reached a loss to follow-up of 10%[35 36] and no data provided for one[34]. As blinding is impossible, it is not considered for downgrading.  2. No difference found in three trials[33 35 36] while one trial found significant difference[34]  5. Numerical data are lacking in 2 trials [34 36] | Very low |
| **Reported symptoms within the first week**  OR (95% CI): 0.95 (0.41-2.20)  *Early discharge versus conventional discharge*  Ramirez-Villalobos 2009[3] | 1 cohort study | -1 | -1 | -1 | -1 | -1 | 1. Large amount of loss of follow-up  2. Only one study  3. the more healthy mother and child and without social issue were included in the early discharge group  4. Large CI including both benefit and harm  5. Assessment of the outcome by interview (recall bias) | Very low |
| **Counseling**  Advices required  For mother: ED 72.6% vs CS 63.0%  For newborn: ED 72.5% vs CS 62.8%  Hellman 1962[34] | 1 RCT | 0 | -1 | 0 | 0 | 0 | 2. Only one RCT | Moderate |
| **Readmission rate**  RR (95% CI): 1.25 (0.54-2.88)  Carty 1990, Hellman 1962, Sainz-Bueno 2005, Smith-Hanrahan 1995, Waldenström 1987, Yanover 1976[33-35 37 38] | 6 RCT | -1 | 0 | 0 | -1 | 0 | 1. Four trials reached a loss to follow-up ≥ 10%[35-38] and no data provided for one[34]. As blinding is impossible, it is not considered for downgrading.  4. CI both includes benefit and harm | Low |
| **Depression**  Carty 1990, Sainz-Bueno 2005, Waldentröm 1987 [33 35 38] | 3 RCT | -1 | 0 | -1 | 0 | 0 | 1. Two trials lost to follow-up ≥ 10% of participants[35 38]. As blinding is impossible, it is not considered for downgrading.  3. Different scales used in both trials | Low |
| **Competence in mothering**  Mean difference (95% CI) at 1 month:  4.3 (-7.7 - 16.3)  Gagnon 1997[39] | 1 RCT | -1 | -1 | 0 | 0 | 0 | 1. Large number of loss to follow-up, required sample size not achieved  2. Only one RCT | Low |
| **Confidence in mothering**  Mean (95% CI) at one week  Group 1: 39.71 (4.68), group 2: 38.73 (5.12), group 3 36.53 (5.83), p<0.03 (group 1 and 3)  Carty 1990[33] | 1 RCT | -1 | -1 | 0 | 0 | 0 | 1. Small sample size  2. Only one RCT | Low |
| **Alliance Scale for negative contact with child**  Mean (SD)  ED: 1.4 (0.53) vs CS: 1.3 (0.45), p=0.661  Akelsdottir 2013[2] | 1 case control study | 0 | -1 | -1 | 0 | 0 | 2. Only one study  3. random sampling for control but convenient sampling for the case | Very low |
| **Alliance Scale for negative contact with father**  Mean (SD)  ED: 1.5 (0.74) vs CS: 1.5 (0.84), p=0.888  Akelsdottir 2013[2] | 1 case control study | 0 | -1 | -1 | 0 | 0 | 2. Only one study  3. random sampling for control but convenient sampling for the case | Very low |
| **Alliance Scale for breast feeding strain**  Mean (SD)  ED: 2.4 (1.31) vs CS: 1.7 (0.93), p=0.001  Akelsdottir 2013[2] | 1 case control study | 0 | -1 | -1 | 0 | 0 | 2. Only one study  3. random sampling for control but convenient sampling for the case | Very low |
| **Alliance Scale for breast feeding uncomfortable**  Mean (SD)  ED: 2.9 (1.61) vs CS: 2.2 (1.07), p=0.028  Akelsdottir 2013[2] | 1 case control study | 0 | -1 | -1 | 0 | 0 | 2. Only one study  3. random sampling for control but convenient sampling for the case | Very low |
| **Alliance Scale for sad mood**  Mean (SD)  ED: 2.0 (0.88) vs CS: 2.1 (0.91), p=0.797  Akelsdottir 2013[2] | 1 case control study | 0 | -1 | -1 | 0 | 0 | 2. Only one study  3. random sampling for control but convenient sampling for the case | Very low |
| **Parent’s Postnatal Sense of Security Scale for empowering behaviour**  Mean (SD)  ED: 3.8 (0.38) vs CS: 3.5 (0.49), p<0.001  Akelsdottir 2013[2] | 1 case control study | 0 | -1 | -1 | 0 | 0 | 2. Only one study  3. random sampling for control but convenient sampling for the case | Very low |
| **Parent’s Postnatal Sense of Security Scale for empowering behaviour**  Mean (SD)  ED: 3.2 (0.55) vs CS: 3.2 (0.74), p=0.341  Akelsdottir 2013[2] | 1 case control study | 0 | -1 | -1 | 0 | 0 | 2. Only one study  3. random sampling for control but convenient sampling for the case | Very low |
| **Parent’s Postnatal Sense of Security Scale for general well-being**  Mean (SD)  ED: 3.2 (0.55) vs CS: 3.2 (0.74), p=0.341  Akelsdottir 2013[2] | 1 case control study | 0 | -1 | -1 | 0 | 0 | 2. Only one study  3. random sampling for control but convenient sampling for the case | Very low |
| **Parent’s Postnatal Sense of Security Scale for affinity in the family**  Mean (SD)  ED: 3.6 (0.48) vs CS: 3.7 (0.43), p=0.886  Akelsdottir 2013[2] | 1 case control study | 0 | -1 | -1 | 0 | 0 | 2. Only one study  3. random sampling for control but convenient sampling for the case | Very low |
| **Parent’s Postnatal Sense of Security Scale for breast feeding**  Mean (SD)  ED: 3.4 (0.65) vs CS: 3.5 (0.63), p=0.644  Akelsdottir 2013[2] | 1 case control study | 0 | -1 | -1 | 0 | 0 | 2. Only one study  3. random sampling for control but convenient sampling for the case | Very low |
| **Neonatal health outcomes** | | | | | | | | |
| **Mortality rate at week 3**  ED 0.24% vs CS 0.46%, ns  Hellman 1962[34] | 1 RCT | 0 | -1 | 0 | 0 | 0 | 1. Not evaluable  2. Only one RCT | Moderate |
| **Need for health contact within 10 days**  RR (95% CI) 0.93 (0.49-1.77)  Carty 1990, Gagnon 1997[33 39] | 2 RCT | 0 | 0 | 0 | -1 | 0 | 4. CI both includes benefit and harm | Moderate |
| **Weight gain**  Gagnon 1997, Hellman 1962[34 39] | 2 RCT | 0 | 0 | -1 | -2 | 0 | 2. Not evaluable  3. Different time frame for the evaluation of outcome  4. No numerical data available for one trial[34] | Very low |
| **Significant neonatal hyperbilirubinemia**  RR (95% CI) 0.50 (0.10-2.50)  Gagnon 1997[39] | 1 RCT | -1 | -1 | 0 | 0 | 0 | 1. Large number of loss to follow-up, required sample size not achieved  2. Only one RCT | Low |
| **Neonatal readmissions rate within 8 weeks**  RR (95% CI) 124 (0.46-3.32)  Hellman 1962, Sainz Bueno 2005, Smith-Hanrahan 1995, Waldenström 1987, Yanover 1976[34 35 37 38] | 5 RCT | -1 | -1 | 0 | -1 | 0 | 1. Four trials reached a loss to follow-up ≥ 10%[35-38] and no data provided for one[34]. As blinding is impossible, it is not considered for downgrading.  2. Point estimates vary widely across studies  4. CI both includes benefit and harm | Very low |
| **Breastfeeding** | | | | | | | | |
| **Breastfeeding within 2 months**  RR (95% CI) 1.10 (0.87-1.40)  Carty 1990, Gagnon 1997, Hellman 1962, Sainz-Bueno 2005[33-39] | 4 RCT | -1 | -1 | 0 | -1 | 0 | 1. Three trials lost to follow-up ≥10% of participants [35 37 38]. As blinding is impossible, it is not considered for downgrading.  2. Large I²  4. CI both includes benefit and harm | Very low |
| **Breastfeeding 1 month postpartum**  ED: 39/43 vs CS: 42/44, p=0.316  Akelsdottir 2013[2] | 1 case control study | 0 | -1 | -1 | 0 | 0 | 2. Only one study  3. random sampling for control but convenient sampling for the case | Very low |
| **Breastfeeding 3 month postpartum**  ED: 29/43 vs CS: 40/44, p=0.021  Akelsdottir 2013[2] | 1 case control study | 0 | -1 | -1 | 0 | 0 | 2. Only one study  3. random sampling for control but convenient sampling for the case | Very low |
| **Breastfeeding 6 month postpartum**  ED: 28/49 vs CS: 20/52, p=0.074  Waldenström 1987[38] | 1 RCT | -1 | -1 | -1 | 0 | 0 | 1. Loss to follow-up = 36.6%  2. Only one study  3. Low sample size | Very low |

1: Limitation, 2: Inconsistency, 3: Indirectness, 4: Imprecision, 5: reporting bias – ED: early discharge – CS: conventional length of stay

# References

1. Brown S, Small R, Argus B, Davis PG, A K. Early postnatal discharge from hospital for healthy mothers and term infants. The Cochrane database of systematic reviews 2009(3. Art. No.: CD002958) doi: 10.1002/14651858.CD002958[published Online First: Epub Date]|.

2. Askelsdottir B, Lam-de Jonge W, Edman G, Wiklund I. Home care after early discharge: impact on healthy mothers and newborns. Midwifery 2013;29(8):927-34

3. Ramirez-Villalobos D, Hernandez-Garduno A, Salinas A, et al. Early hospital discharge and early puerperal complications. Salud Publica de Mexico 2009;51(3):212-8

4. Almond D, Doyle JJ, Jr., Kowalski AE, Williams H. Estimating Marginal Returns to Medical Care: Evidence from At-Risk Newborns. Quarterly Journal of Economics 2010;125(2):591-634

5. Barker K. Cinderella of the services - 'the pantomime of postnatal care'. British Journal of Midwifery 2013;21(12):842-42

6. Batu E.D, Yeni S, Teksam O. The factors affecting neonatal presentations to the pediatric emergency department. Journal of Emergency Medicine 2015;48(5):542-47

7. Benitz WE, Committee on F, Newborn AAoP. Hospital stay for healthy term newborn infants. Pediatrics 2015;135(5):948-53

8. Bernstein HH, Spino C, Lalama CM, Finch SA, Wasserman RC, McCormick MC. Unreadiness for Postpartum Discharge Following Healthy Term Pregnancy: Impact on Health Care Use and Outcomes. Academic Pediatrics 2013;13(1):27-39

9. Boulvain M, Perneger TV, Othenin-Girard V, Petrou S, Berner M, O I. Home-based versus hospital-based postnatal care: a randomised trial. BJOG: an international journal of obstetrics and gynaecology 2004;111:807–13

10. Bravo P, Uribe C, Contreras A. Early postnatal hospital discharge: the consequences of reducing length of stay for women and newborns. Revista Da Escola de Enfermagem Da Usp 2011;45(3):758-63

11. Brooten D, Roncoli M, Finkler S, Arnold L, Cohen A, M M. A randomized trial of early hospital discharge and home follow up of women having caesarean birth. Obstetrics & Gynecology 1994;84(5):832-8

12. Cambonie G, Rey V, Sabarros S, et al. Early postpartum discharge and breastfeeding: an observational study from France. Pediatrics International 2010;52(2):180-6

13. Chanot A, Semet JC, Arnaud C, et al. L'hospitalisation a domicile apres une sortie precoce de maternite. Soins 2009;Pediatrie, Puericulture.(246):24-6

14. Chanot AA, Semet JC, Arnaud C, et al. Sorties precoces de maternite et hospitalisation a domicile: experience ariegeoise. Archives de Pediatrie 2009;16(6):706-8

15. Cuncarr C, Skinner J. Quantity or quality of postnatal length of stay? A literature review examining the issues and the evidence. New Zealand College of Midwives Journal 2011(44):12-16

16. Das JK, Kumar R, Salam RA, Lassi ZS, Bhutta ZA. Evidence from facility level inputs to improve quality of care for maternal and newborn health: interventions and findings. Reproductive health 2014;11(2):4 doi: Source: NLM. PMC4160922[published Online First: Epub Date]|.

17. De Carolis MP, Cocca C, Valente E, et al. Individualized follow up programme and early discharge in term neonates. Italian journal of pediatrics 2014;40(70) doi: Source: NLM. PMC4223512[published Online First: Epub Date]|.

18. De Luca D, Carnielli VP, Paolillo P. Neonatal hyperbilirubinemia and early discharge from the maternity ward. European Journal of Pediatrics 2009;168(9):1025-30

19. Evans WN, Garthwaite C, Wei H, Nber. The impact of early discharge laws on the health of newborns. Journal of Health Economics 2008;27(4):843-70

20. Farhat R, Rajab M. Length of postnatal hospital stay in healthy newborns and re-hospitalization following early discharge. North American Journal of Medical Sciences 2011;3(3):146-51

21. Fink AM. Early hospital discharge in maternal and newborn care. JOGNN - Journal of Obstetric, Gynecologic, & Neonatal Nursing 2011;40(2):149-56

22. Forster DA, Savage TL, McLachlan HL, et al. Individualised, flexible postnatal care: a feasibility study for a randomised controlled trial. BMC Health Services Research 2014;14(1):569 doi: Source: NLM. PMC4279591[published Online First: Epub Date]|.

23. Ghedin B.S, Cancelier A.C.L, Feuerschuette O.H.M, Silveira S.K. Healthy newborn length of hospital stay: Influence on breastfeeding and neonatal morbidity. Journal of Perinatal Medicine 2011;39

24. Gotink MJ, Benders MJ, Lavrijsen SW, Rodrigues Pereira R, Hulzebos CV, Dijk PH. Severe neonatal hyperbilirubinemia in the Netherlands. Neonatology 2013;104(2):137-42

25. Houghton S. Research round-up. Mental Health Practice 2008;11(5):21-21

26. Lain SJ, Roberts CL, Bowen JR, Nassar N. Early discharge of infants and risk of readmission for jaundice. Pediatrics 2015;135(2):314-21

27. McIntosh B, Bewley S. How far can a complex system with increased interventions be pushed? British Journal of Midwifery 2012;20(3):170-72

28. Skene C, Gupta A, Flaherty M, Sherwood E. Neonatal rapid access clinic: an innovative practice to reduce neonatal readmissions to hospital. Infant 2012;8(6):184-86

29. Soares da NÃ³brega Mazzo MH, Santana de Brito R, Lima de Souza N, Pires Gama A. Taking Care of the Puerpera after Hospital Discharge: A Literature Review. Journal of Nursing UFPE / Revista de Enfermagem UFPE 2012;6(11):2823-29 doi: 10.5205/reuol.2185-16342-1-LE.0611201227[published Online First: Epub Date]|.

30. Straczek H, Vieux R, Hubert C, Miton A, Hascoet JM. Sorties precoces de maternite : quels problemes anticiper ? Archives de Pediatrie 2008;15(6):1076-82

31. Winterburn S, R F. Does the duration of postnatal stay influence breast-feeding rates at one month in women giving birth for the first time? A randomized control trial. Journal of Advanced Nursing 2000;32(5):1152–7

32. Yonemoto N, Dowswell T, Nagai S, Mori R. Schedules for home visits in the early postpartum period. The Cochrane database of systematic reviews 2013;7

33. Carty E, C B. A randomized, controlled evaluation of early postpartum hospital discharge. Birth 1990;17(4):199–204

34. Hellman L.M., Kohl S.G. Early hospital discharge in obstetrics. Lancet 1962;1(227-33)

35. Sainz Bueno JA, Romano MR, Teruel RG, et al. Early discharge from obstetrics-pediatrics at the Hospital de Valme, with domiciliary follow-up. American Journal of Obstetrics and Gynecology 2005;193(3 Pt 1):714–26

36. Yanover MJ, Jones D, MD M. Perinatal care of low-risk mothers and infants. Early discharge with home care. New England Journal of Medicine 1976;294(13):702–5

37. Smith-Hanrahan C, D D. Postpartum early discharge: impact of maternal fatigue and functional ability. Clinical Nursing Research 1995;4(1):50-66

38. Waldenström U, G. L. Early and late discharge after hospital birth. A comparative study of parental background characteristics. Scandinavian Journal of Social Medicine 1987;15:159–67

39. Gagnon A, Edgar L, Kramer M, Papageorgiou A, Waghorn K, M K. A randomized trial of a program of early postpartum discharge with nurse visitation. American Journal of Obstetrics and Gynecology 1997;176(1):205-11
